# Investigating the Propagation Mechanisms and Visualization of Airwaves in Marine CSEM Using the Fictitious Wave Domain Method

**DOI:** 10.3390/s25237140

**Published:** 2025-11-22

**Authors:** Jie Lu, Daicheng Peng

**Affiliations:** 1 Key Laboratory of Exploration Technologies for Oil and Gas Resources of MOE, Yangtze University, Wuhan 430100, China; 2 College of Geophysics and Petroleum Resources, Yangtze University, Wuhan 430100, China; 3 Sinopec Petroleum Engineering Geophysics Co., Ltd. Wuhan Exploration Branch, Qianjiang 433100, China

**Keywords:** marine CSEM, airwave, diffusive frequency domain, fictitious wave domain, reflection, refraction, travel time, signal separation

## Abstract

**Highlights:**

**What are the main findings?**

- The low-frequency diffusive electromagnetic field generated by an electric dipole in marine environments can be mathematically transformed into corresponding fictitious electromagnetic waves in the fictitious wave domain. These waves exhibit no physical energy attenuation, allowing both their kinematic and dynamic characteristics to be used for analyzing the propagation behavior of low-frequency EM fields.
- Using the propagation features of fictitious electromagnetic waves, the generation mechanism, propagation characteristics, and extraction method of the airwave in marine CSEM surveys are reinterpreted.

**What is the implication of the main findings?**

- The fictitious wave domain method provides a novel perspective for analyzing and interpreting various electromagnetic phenomena in marine CSEM surveys by leveraging both the kinematic and dynamic attributes of fictitious electromagnetic waves.
- In the fictitious wave domain, EM responses from different target layers can be distinguished and extracted based on travel time differences, enabling more detailed analysis and providing theoretical support for the development of advanced marine electromagnetic exploration sensors and systems.

**Abstract:**

The marine controlled-source electromagnetic (CSEM) method serves as an effective tool for detecting hydrocarbon reservoirs. However, it faces a key challenge in shallow water: the airwave, an EM signal lacking subsurface information, often obscures reservoir responses. Conventional CSEM analysis, conducted in the diffusive frequency domain (DFD), only captures the steady-state behavior of the airwave, limiting physical insight into its propagation. In this study, we introduce the fictitious wave domain (FWD) method to reinterpret and visualize the airwaves’ trajectory and attenuation, individually. By transforming diffusive EM fields into fictitious lossless propagating waves, the FWD enables the use of kinematic wave concepts such as reflection, refraction, and travel time. The airwave is clearly identified as a refracted wave generated when a transverse electromagnetic (TEM) mode wave impinges perpendicularly on the air–seawater interface. Its path and arrival time become directly observable, allowing clear separation from other wave types. This approach visualizes and extracts the airwave even in complex inhomogeneous seawater, enabling its accurate transformation back to the DFD. The FWD thus provides a powerful tool for enhancing interpretation in marine EM exploration and offers a theoretical foundation for the development of tailored marine electromagnetic sensors.

## 1. Introduction

The marine controlled-source electromagnetic (CSEM) method, pioneered by Cox [1], has become a cornerstone technology for offshore hydrocarbon exploration. In a typical CSEM survey, a detection vessel tows an electric dipole approximately 100–200 m in length, which transmits alternating current at frequencies ranging from 0.1 Hz to 10 Hz. Meanwhile, receivers equipped with electric and magnetic field sensors are deployed on the seafloor to record the alternating electromagnetic fields induced in the subsurface by the transmitted signal [2]. This approach has demonstrated significant efficacy in deep-water environments [3] and has yielded remarkable achievements, garnering widespread recognition within the industry [4]. However, in shallow-water environments, the method encounters a critical limitation: electromagnetic interactions with the air layer generate a dominant airwave that severely masks subsurface signals, particularly the responses from thin high-resistivity reservoirs at large source-receiver offsets [5,6]. This airwave, characterized as a pure transverse electric (TE) mode wave with a distinct $1/r^{3}$-decay behavior [7], propagates along the air-seawater interface and contains no information about subsurface reservoirs, fundamentally impeding the detection of hydrocarbon-bearing formations in shallow-water surveys.

To enhance the feasibility of shallow-water marine CSEM surveys, numerous strategies have been developed to mitigate or eliminate airwave interference. Although the use of vertical dipole sources or vertical field components avoids airwave effects in theory [8], these methods face substantial practical challenges. An alternative involves decomposing the marine CSEM response into upgoing and downgoing wavefields: the downgoing wave contains the airwave, while the upgoing wave carries the response of high-resistivity targets. Analyzing the upgoing component thus eliminates airwave contamination [9,10,11].

Further mitigation leverages wave mode polarization: the airwave manifests as a pure transverse electric (TE) wave, whereas reservoir signals exhibit transverse magnetic (TM) characteristics. Decomposing the response and removing the TE component effectively suppresses airwave effects, even for models with uneven seafloor topography [5]. Additionally, airwave separation can exploit distinct dependencies on offset [12,13] and frequency [13,14] relative to subsurface signals. By applying weighted differential fields to marine controlled-source electromagnetic data, Li et al. effectively suppressed the airwave effect in shallow waters, enabling improved detection of gas hydrates [15].

Bannister [16] and Weidelt [7] established the theoretical formulation for pure airwave responses, while Nordskag and Amundsen [17] explicitly incorporated the effects of airwave reflections and reverberations within an infinite seawater layer. However, these formulations are restricted to 1D homogeneous seawater models and characterize only the time-harmonic steady-state behavior of the air wave.

The Fictitious Wave Domain (FWD) method, first developed by Lee et al. [18], de Hoop [19], and Mittet [20], enables computation of electromagnetic (EM) responses in the frequency domain through mathematical transformation of fictitious EM waves. Within this framework, fictitious EM waves propagate without attenuation, allowing intuitive application of reflection, transmission, and refraction concepts for interpretation and analysis. Mittet [20,21] demonstrated that the propagation characteristics of fictitious EM waves exhibit strong analogies to seismic wave behavior. Subsequently, Lu et al. [22] established that the frequency-domain airwave corresponds to the Air Refraction Wave (ARW) in the FWD, analyzing its performance in canonical reservoir models for both deep-water and shallow-water environments. However, the models considered by Lu et al. [22], while canonical, incorporated sufficient complexity such that the ARW (representing the airwave) remained significantly influenced by other interfering wave components.

It is evident that, in contrast to the FWD approach, previous studies on the airwave have primarily been conducted in the frequency domain, focusing on its steady-state behavior and its distinguishing features compared to signals originating from subseabed formations—often under the assumption of a homogeneous conductive seawater medium. However, within the FWD framework, both the airwaves and other wave types exhibit distinct propagation paths and arrival times. Their transmission and attenuation processes can be visually reconstructed, making the characteristics of the airwave more straightforward to analyze and interpret.

Building upon the foundational work of Mittet [20,21] and Lu et al. [22], this paper systematically investigates the generation and propagation characteristics of the pure airwave in both whole-space and half-space seawater models. Leveraging the distinct traveltime differences between the airwave and subsurface electromagnetic signals, we propose a novel airwave extraction and quantification method within the FWD. Crucially, this approach is effective not only in seawater with homogeneous conductivity but also extends to realistic inhomogeneous seawater environments. By examining the fundamental electromagnetic interaction between a marine electric dipole and the air-seawater interface, this research establishes a theoretical basis for marine CSEM detection and underwater electromagnetic communication.

## 2. Theory

### 2.1. Fictitious Wave Domain Method

Ignoring the displacement current and assuming an $e^{-i\omega t}$ time dependence, the quasi-static frequency-domain Maxwell’s equations are:

(1) $$\nabla \times H\left(\omega \right)-\sigma E\left(\omega \right)=J\left(\omega \right),$$

(2) $$\nabla \times E\left(\omega \right)-i\omega \mu H\left(\omega \right)=0,$$

where E and H denote the electric and magnetic vector fields, respectively, and the source term J represents the external electric current density. The electrical conductivity is denoted by σ, while the magnetic permeability, μ, is set to that of free space. The angular frequency ω is defined within the diffusive frequency domain (DFD).

The EM phase velocity c in the diffusive domain is given by:

(3) $$c=\sqrt{\frac{2\omega }{\mu \sigma }}.$$

Two parameters are defined here: $\varepsilon^{\prime }$, the fictitious dielectric permittivity, and $\omega^{\prime }$, the fictitious angular frequency [20].

(4) $$\varepsilon^{\prime }=\frac{\sigma }{2\omega_{0}},$$

(5) $$\omega^{\prime }=\left(i+1\right)\sqrt{\omega \omega_{0}},$$

where $\omega_{0}$ is an arbitrary positive real number, designated as the transformation factor. Owing to its analogous role to ω in the equations, it is also termed the fictitious angular frequency, and is defined by the relation $\omega_{0}=2\pi f_{0}$, where $f_{0}$ denotes the fictitious frequency.

By means of the variable substitutions given in Equations (4) and (5), Maxwell’s equations in Equations (1) and (2) in the DFD can be reformulated in terms of a fictitious wave frequency domain as follows:

(6) $$\nabla \times H^{\prime }\left(\omega^{\prime }\right)+i\omega^{\prime }\varepsilon^{\prime }E^{\prime }\left(\omega^{\prime }\right)=J^{\prime }\left(\omega^{\prime }\right),$$

(7) $$\nabla \times E^{\prime }\left(\omega^{\prime }\right)-i\omega^{\prime }\mu H^{\prime }\left(\omega^{\prime }\right)=0,$$

The variable substitutions are defined as follows: $E^{\prime }\left(\omega^{\prime }\right)=E\left(\omega \right)$, $H^{\prime }\left(\omega^{\prime }\right)=\sqrt{\frac{-i\omega }{2\omega_{0}}}H\left(\omega \right)$, $J^{\prime }\left(\omega^{\prime }\right)=\sqrt{\frac{-i\omega }{2\omega_{0}}}J\left(\omega \right)$. Here $E^{\prime }$ and $H^{\prime }$ are the fictitious electric and magnetic vector fields, respectively, and $J^{\prime }$ is the corresponding fictitious external electric current density.

Maxwell’s equations in the FWD are defined as the inverse Fourier transform of their frequency-domain counterparts:

(8) $$\nabla \times H^{\prime }\left(t^{\prime }\right)-\varepsilon^{\prime }\frac{\partial E^{\prime }\left(t^{\prime }\right)}{\partial t^{\prime }}=J^{\prime }\left(t^{\prime }\right),$$

(9) $$\nabla \times E^{\prime }\left(t^{\prime }\right)+\mu \frac{\partial H^{\prime }\left(t^{\prime }\right)}{\partial t^{\prime }}=0,$$

Unlike Equation (3), the fictitious EM wave group and phase velocity $c^{\prime }$ is finite and solely medium-dependent.

(10) $$c^{\prime }=\sqrt{\frac{1}{\mu \varepsilon^{\prime }}}=\sqrt{\frac{2\omega_{0}}{\mu \sigma }}.$$

The fictitious EM wave velocity is governed by the transformation factor ω_0_ and the conductivity. Thus, for a given conductive model, an appropriate wave velocity can be selected by adjusting $\omega_{0}$ to achieve an optimal balance between computational accuracy and efficiency.

Once Equations (8) and (9) are solved using the numerical method, the resulting fictitious EM fields and current density within the fictitious time range from 0 to $T^{\prime }$ can then be transformed back to the diffusive frequency domain.

(11) $$E\left(\omega \right)=\int_{0}^{T^{\prime }}E^{\prime }\left(t^{\prime }\right)e^{-\sqrt{\omega \omega_{0}}t^{\prime }}e^{i\sqrt{\omega \omega_{0}}t^{\prime }}dt^{\prime },$$

(12) $$H\left(\omega \right)=\sqrt{\frac{2\omega_{0}}{-i\omega }}\int_{0}^{T^{\prime }}H^{\prime }\left(t^{\prime }\right)e^{-\sqrt{\omega \omega_{0}}t^{\prime }}e^{i\sqrt{\omega \omega_{0}}t^{\prime }}dt^{\prime },$$

(13) $$J\left(\omega \right)=\sqrt{\frac{2\omega_{0}}{-i\omega }}\int_{0}^{T^{\prime }}J^{\prime }\left(t^{\prime }\right)e^{-\sqrt{\omega \omega_{0}}t^{\prime }}e^{i\sqrt{\omega \omega_{0}}t^{\prime }}dt^{\prime },$$

The time-domain excitation for the external current density in FWD is defined as the first derivative of a Gaussian function:

(14) $$J^{\prime }\left(t^{\prime }\right)=-2\beta \left(t^{\prime }-t_{0}\right)\sqrt{\frac{\beta }{\pi }}e^{-\beta {\left(t^{\prime }-t_{0}\right)}^{2}},$$

where $\beta =\pi f_{max}^{2}$ and $t_{0}=\pi /f_{max}$. $f_{max}$ is the frequency from the dispersion analysis [20].

The kernel of the modified Fourier transformation in Equations (11)–(13) is the damping factor $e^{-\sqrt{\omega \omega_{0}}t^{\prime }}$, which governs the mapping from the FWD to the DFD through two complementary mechanisms: (1) Fixed ω: The factor decays with fictitious time $t^{\prime }$, acting as a temporal window that emphasizes early-arriving waves and suppresses later ones in the construction of the DFD response. This imposes a causal priority on the early portion of the wavefield. (2) Varying ω: The same factor causes exponential amplitude decay with increasing ω, mirroring the diffusive nature of the DFD, where high-frequency components are naturally attenuated. Concurrently, the stronger damping at high frequencies effectively increases the relative contribution of early arrivals to the synthesized response, leading to a frequency-dependent weighting of the fictitious wavefield’s temporal evolution.

The core of the FWD method is to mask EM attenuation. It first introduces a fictitious dielectric constant $\varepsilon^{\prime }$ to recast the conductive medium as a dielectric, and then uses a complex angular frequency $\omega^{\prime }$ to hide the energy loss. The field’s actual attenuation is recovered later by the inverse Fourier transform.

The FWD method effectively decouples the traditional solution process of Equations (1) and (2) into two independent stages: (1) Solving Equations (8) and (9), which describe the propagation of fictitious EM waves without attenuation. (2) Calculating the frequency-domain CSEM response by transforming the fictitious wavefields using Equations (11)–(13), which incorporate the necessary time- and frequency-dependent attenuation. In this framework, the wave propagation process depends solely on the geological model and survey configuration, while the attenuation and integration process is determined by the specified exploration frequencies. This clear separation between propagation and attenuation facilitates more flexible and efficient simulation. Fundamental analyses and applications of this decoupling have been discussed by Mittet [21] and Lu et al. [22].

### 2.2. Dipole Source in Whole-Space Seawater

In the FWD, the electric signal exhibits a wavelet-like character, distinctly different from the diffusive behavior in the DFD. The propagation of the fictitious EM wave from a dipole source in a homogeneous whole space serves as the foundational case for understanding wave behavior in more complex models. A horizontal electric dipole (HED) is a universal example, as sources of arbitrary dip angles can be derived through coordinate rotation from an HED. Here, an x-directed HED is placed at the origin (0,0,0). The XZ plane represents the inline-mode plane, commonly used in practical surveys, where only the components ${E_{x}}^{\prime }$, ${E_{z}}^{\prime }$ and ${H_{y}}^{\prime }$ are non-zero.

**Figure 1:**
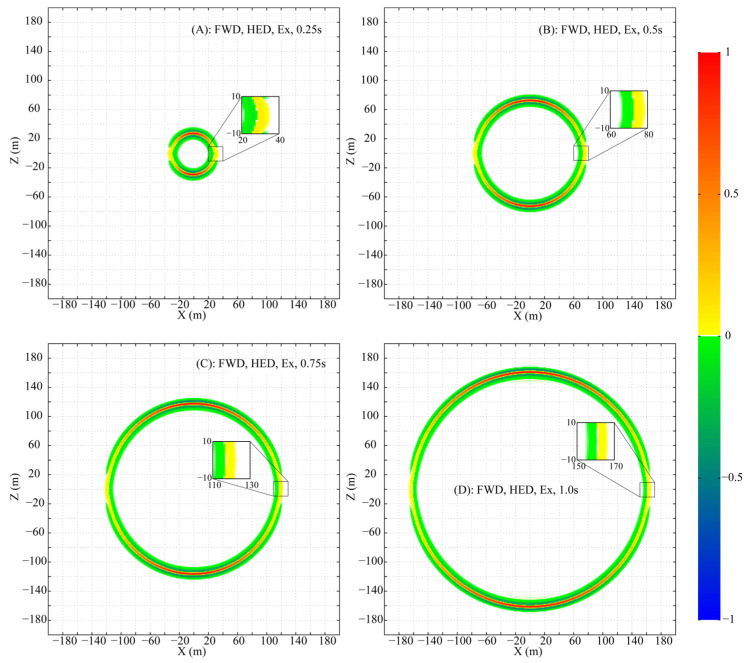
Snapshots of the fictitious wavefield component ${E_{x}}^{\prime }$ in the XZ plane at selected times (All amplitudes are normalized to their respective in-plane maxima).

**Figure 2:**
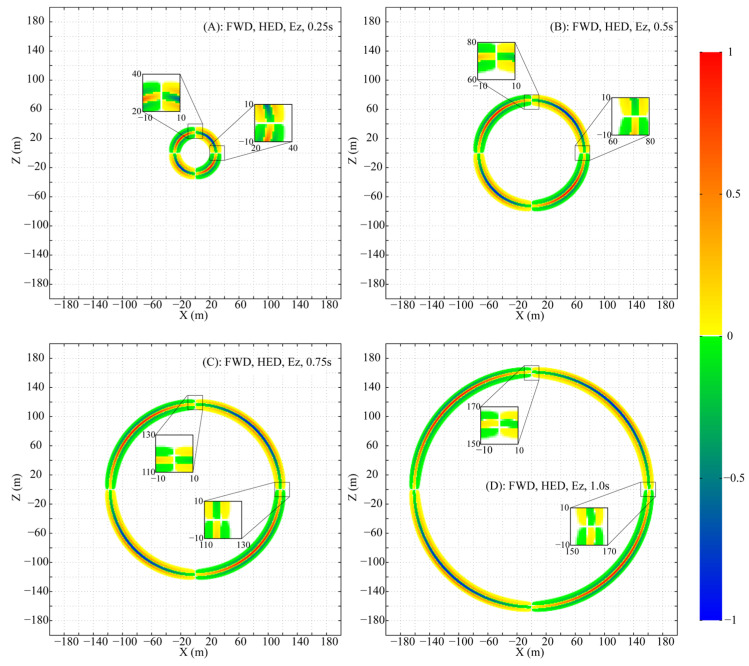
Snapshots of the fictitious wavefield component ${E_{z}}^{\prime }$ in the XZ plane at selected times (All amplitudes are normalized to their respective in-plane maxima).

**Figure 3:**
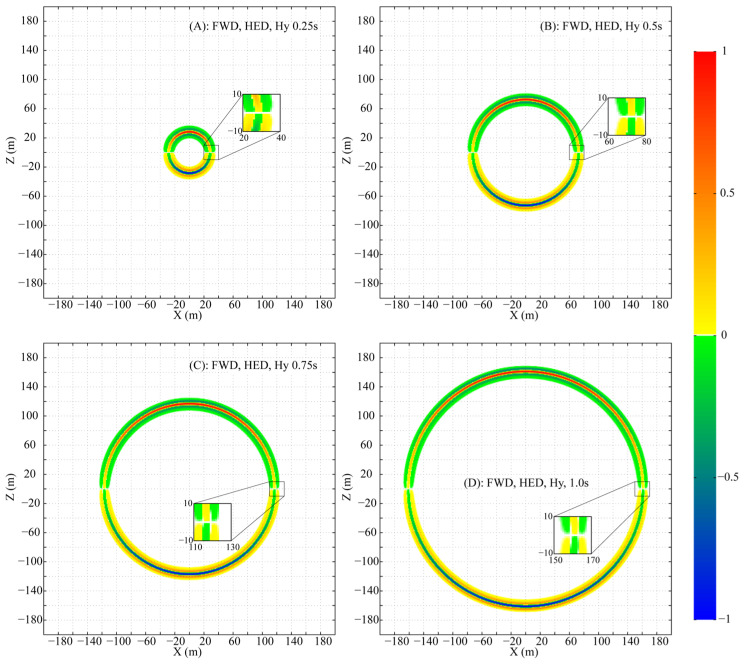
Snapshots of the fictitious wavefield component ${H_{y}}^{\prime }$ in the XZ plane at selected times (All amplitudes are normalized to their respective in-plane maxima).

To meticulously analyze the propagation, the parameters are set as follows: $f_{0}=0.01$, $f_{max}=3.5$, and a uniform mesh size of 1 m×1 m×1 m is adopted to ensure that numerical dispersion is maintained below 0.1%. Snapshots of the normalized wavefields for ${E_{x}}^{\prime }$, ${E_{z}}^{\prime }$ and ${H_{y}}^{\prime }$ at times 0.25 s, 0.50 s, 0.75 s, and 1.00 s are presented in Figure 1, Figure 2, and Figure 3, respectively. For clear visualization, the amplitude of each component at each time step is normalized by its respective maximum absolute value across the entire plane, thereby isolating the wave patterns from the effects of geometric attenuation.

The HED, being a 2D source, differs from a 3D point source. Nevertheless, the propagation of its fields (${E_{x}}^{\prime }$, ${E_{z}}^{\prime }$ and ${H_{y}}^{\prime }$) in the XZ plane forms a circular-like pattern. Despite this geometric similarity, the amplitude and phase vary significantly along the circumference of the propagation circle.

${E_{x}}^{\prime }$ peaks in amplitude directly above the HED and decays, without vanishing, towards the axial direction (Figure 1). ${E_{z}}^{\prime }$ exhibits a more complex pattern: its amplitude is maximal at azimuths of ~±45° and ±135°, with a phase reversal between the 0°–90°/−90°–−180° and 90°–180°/0°–−90° ranges, and is zero above and along the HED axis (Figure 2). ${H_{y}}^{\prime }$ is strongest above the HED with a sign reversal across the source plane and is identically zero along the entire x-axis at z=0 (Figure 3).

Two special lines in the XZ plane are defined: (A) x=0 and (B) z=0. On line A, the non-zero components ${E_{x}}^{\prime }$ and ${H_{y}}^{\prime }$, with $E_{z}^{\prime }=0$, form a TEM mode. In contrast, on line B, only ${E_{x}}^{\prime }$ is non-zero ($E_{z}^{\prime }=0$, $H_{y}^{\prime }=0$), a field structure that does not conform to standard TE or TM mode definitions.

**Figure 4:**
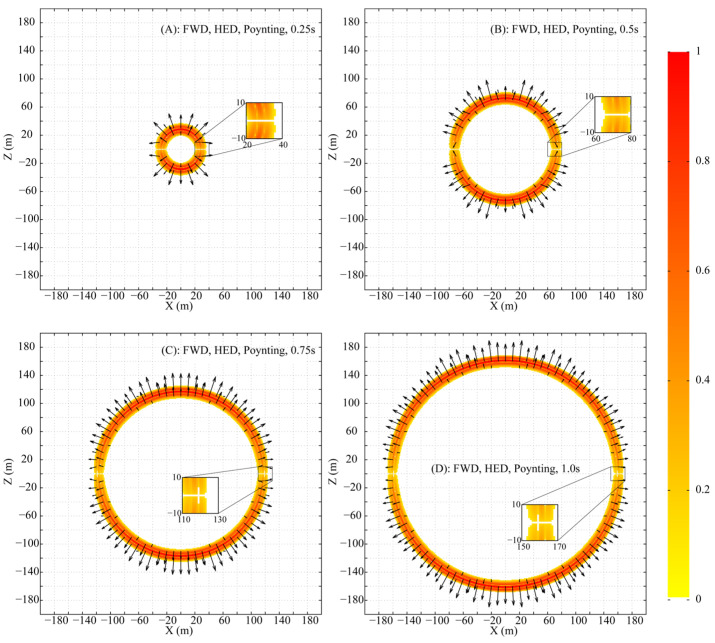
Time snapshots of the fictitious Poynting vector distribution in the XZ plane (Amplitude is normalized to the in-plane maximum; direction is shown by arrows).

The fictitious Poynting vector, defined as $S^{\prime }=E^{\prime }\times H^{\prime }$, is computed from the simulated field data and visualized in Figure 4 (amplitudes normalized, directions shown by arrows). This visualization confirms two key observations: firstly, along the vertical line x=0, the energy propagation is purely vertical. Secondly, and more notably, the Poynting vector is identically zero everywhere along the horizontal line z=0, thereby confirming that the presence of ${E_{x}}^{\prime }$ does not result in any net electromagnetic energy flux in this region.

**Figure 5:**
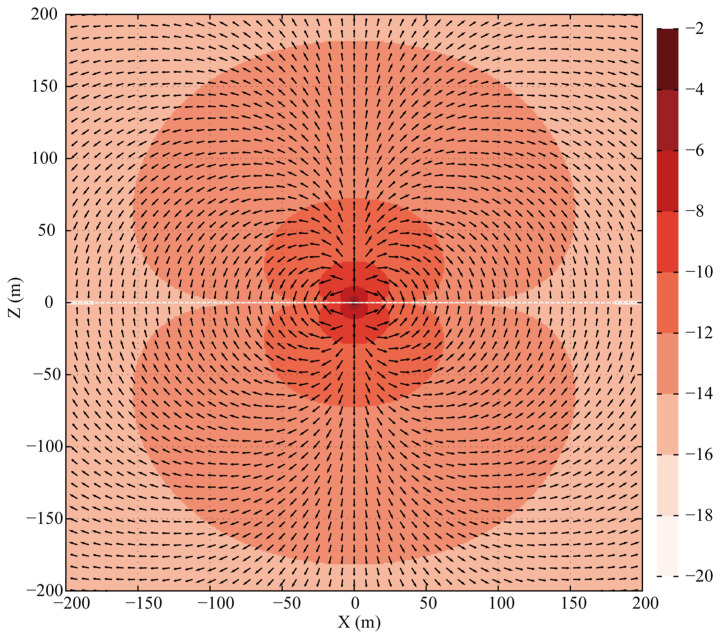
Spatial distribution (log_10_ magnitude) of the real part of the complex Poynting vector for a 1.0 Hz HED at the origin in a whole-space DFD model. Arrows indicate the direction of energy flow.

In the DFD, the time-averaged complex Poynting vector for a whole-space HED can be readily computed, with its real part representing the net energy flux density into a medium. Figure 5 illustrates the spatial distribution of the real part of this Poynting vector at 1.0 Hz. In contrast to the concentric circular pattern observed in the FWD, the magnitude distribution in the DFD exhibits a flattened, dumbbell-like shape (figure-eight pattern). Furthermore, the direction of the time-averaged energy flow is perfectly perpendicular to the HED along the line x=0 and is entirely absent along z=0, which is fully consistent with the propagation characteristics identified in the FWD.

## 3. Airwave in Half-Space Seawater Model

### 3.1. Propagation Path, Reflection, and Transmission at the Air-Seawater Interface

**Figure 6:**
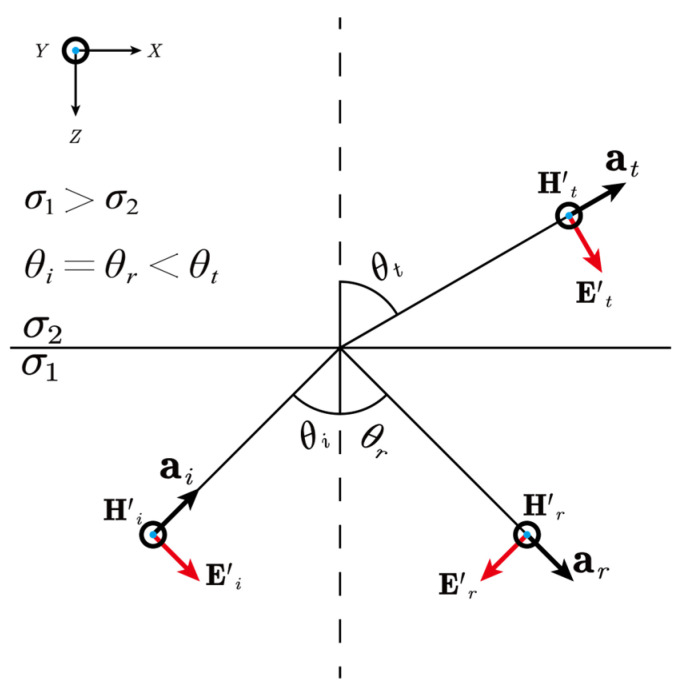
Schematic illustration of a fictitious EM wave incident upon an interface from a high-conductivity to a low-conductivity material. The vectors $E^{\prime }$ and $H^{\prime }$ represent the fictitious electric and magnetic fields, respectively. The subscripts i, r and t denote the incident, reflected, and transmitted wave components; their corresponding angles are $\theta_{i}$, $\theta_{r}$, and $\theta_{t}$. The unit vector a indicates the direction of wave propagation.

Consider a scenario where a fictitious EM wave is incident on an interface from a high-conductivity to a low-conductivity material, as depicted in Figure 6. At the interface, the wave generates both reflected and transmitted components, and the relationship between their propagation angles is governed by Snell’s Law:

(15) $$\frac{sin\theta_{i}}{{c_{1}}^{\prime }}=\frac{sin\theta_{r}}{{c_{1}}^{\prime }}=\frac{sin\theta_{t}}{{c_{2}}^{\prime }},$$

where the subscripts i, r, and t are assigned to the incident, reflected, and transmitted waves, respectively. $\theta_{i}$, $\theta_{r}$ and $\theta_{t}$ are the corresponding angles. The fictitious EM wave velocities ${c_{1}}^{\prime }$ and ${c_{2}}^{\prime }$ in the two media depend on their conductivities, as given by Equation (10), with lower conductivity resulting in higher wave speed. Therefore, in this case, $\theta_{i}=\theta_{r}<\theta_{t}$.

**Figure 7:**
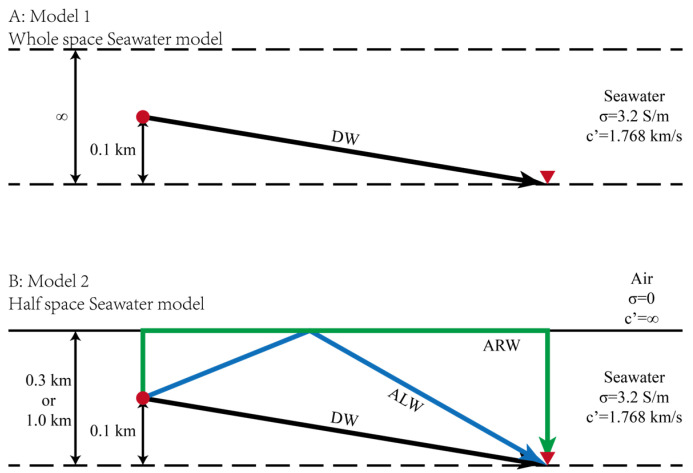
Schematic diagram illustrating the propagation paths of fictitious EM waves in (**A**) a whole-space seawater model and (**B**) a half-space seawater model. **DW**, **ARW,** and **ALW** denote the Direct Wave, Air-refRaction Wave, and Air-refLection Wave, respectively. See the text for detailed explanations.

The propagation of the fictitious EM wave is first analyzed in a whole-space seawater model (conductivity 3.3 S/m, fictitious frequency $f_{0}=1\;Hz$, wave velocity ${c^{\prime }}_{sea}=1.768\;km/s$, Figure 7A). A receiver array is placed $h_{source}=0.1\;km$ beneath the source. Since no interfaces exist, the sole arrival is the **Direct Wave** (**DW**), whose travel time ${t^{\prime }}_{DW}$ relates to the offset s through the hyperbolic equation:

(16) $$\frac{{t^{\prime }}_{DW}^{2}}{\frac{h_{source}^{2}}{{c^{\prime }}_{sea}^{2}}}-\frac{s^{2}}{h_{source}^{2}}=1.$$

Figure 7B depicts a two-layer air-seawater model. Air has zero conductivity and infinite wave velocity, far exceeding seawater’s. With receivers at $h_{sea}=0.3\;km$ (or $h_{sea}=1.0\;km$) below the interface and the source $h_{source}=0.1\;km$ above them, the upgoing wave interacts with the interface. This generates an **Air refLection Wave (ALW)** for $\theta_{i}$ > 0°, and at the critical angle ($\theta_{i}$ = 0°), a special **Air refRaction Wave (ARW)** that travels instantaneously along the interface due to the infinite air velocity. The offset–traveltime relations of **ALW** (${t^{\prime }}_{ALW}$) and **ARW** (${t^{\prime }}_{ARW}$) are:

(17) $$\frac{{t^{\prime }}_{ALW}^{2}}{\frac{{\left(2h_{sea}-h_{source}\right)}^{2}}{{c^{\prime }}_{sea}^{2}}}-\frac{s^{2}}{{\left(2h_{sea}-h_{source}\right)}^{2}}=1$$

(18) $${t^{\prime }}_{ARW}=\frac{2h_{sea}-h_{source}}{{c^{\prime }}_{sea}}$$

Despite the similarity in their travel time curves, the **ALW** always arrives after the **DW** because $h_{sea}$ is invariably greater than $h_{source}$.

### 3.2. Airwave in Homogeneous Half-Space Seawater

**Figure 8:**
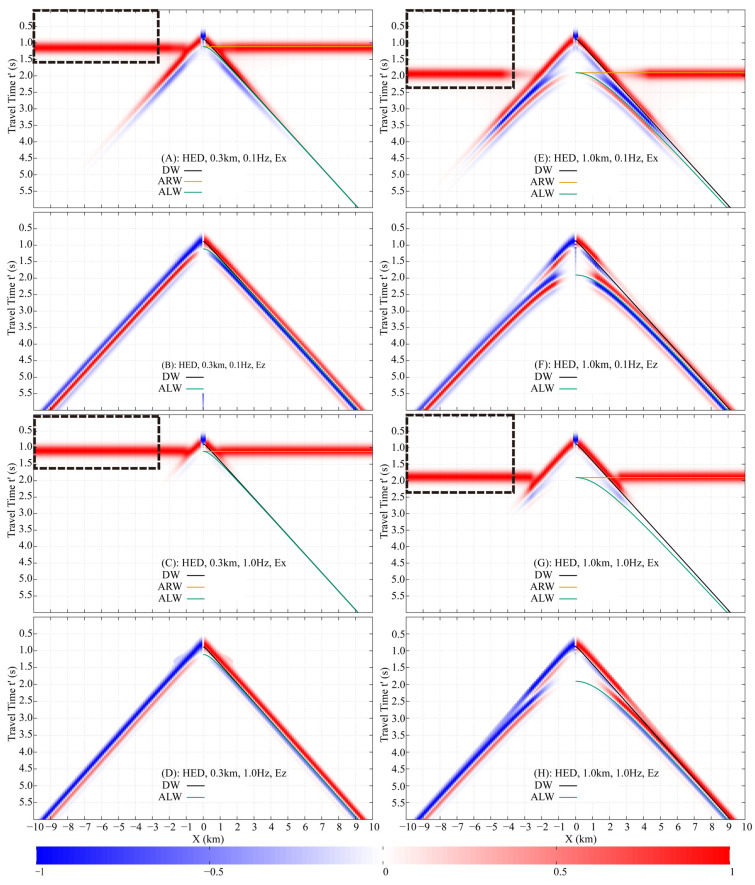
Normalized shot gathers of fictitious electric fields ${E^{\prime }}_{x}$ and ${E^{\prime }}_{z}$, showing responses at receiver depths of 0.3 km and 1.0 km below the air-seawater interface, and frequencies of 0.1 Hz and 1.0 Hz. Theoretical traveltime curves for **DW**, **ALW**, and **ARW** are overlaid. The black dashed rectangle represents the offset for accurate airwave extraction and the upper and lower limits of the fictitious time integration. (**A**) HED, 0.3 km, 0.1 Hz, Ex. (**B**) HED, 0.3 km, 0.1 Hz, Ez. (**C**) HED, 0.3 km, 1.0 Hz, Ex. (**D**) HED, 0.3 km, 1.0 Hz, Ez. (**E**) HED, 1.0 km, 0.1 Hz, Ex. (**F**) HED, 1.0 km, 0.1 Hz, Ex. (**G**) HED, 1.0 km, 1.0 Hz, Ex. (**H**) HED, 1.0 km, 1.0 Hz, Ez.

The Finite-Difference Time-Domain (FDTD) method is used to simulate the fictitious EM wavefields in a half-space seawater model (Figure 7B), with a 0.1 km×0.1 km×0.1 km mesh, $f_{0}=1\;Hz$, $f_{max}=3.5\;Hz$, and offsets from −10 km to 10 km. A 10th-order finite-difference scheme was employed, which maintained numerical dispersion below 0.1%. To satisfy the CFL condition and ensure computational accuracy, a fictitious time step of 0.01 s was selected. A convolutional perfectly matched layer (CPML) absorbing boundary condition was applied, extending 10 grid cells beyond the study area in all directions to host the CPML medium. Results for receiver depths of 0.3 km and 1.0 km are processed into normalized shot gathers (Figure 8), showing ${E^{\prime }}_{x}$ and ${E^{\prime }}_{z}$ at 0.1 Hz and 1.0 Hz, incorporating the damping factor $e^{-\sqrt{\omega \omega_{0}}t^{\prime }}$. Theoretical traveltime curves of the **DW**, **ALW**, and **ARW** are plotted alongside, demonstrating excellent agreement with the numerical data.

The arrival time of the **DW** is solely dependent on the relative position between the source and receivers, not their absolute burial depths. In both the 0.3 km and 1.0 km models, the receivers are positioned 0.1 km directly below the HED source. Consequently, the **DW** arrival times are identical in both cases, as confirmed by the data in Figure 8.

The travel time of the **ALW** is determined by both the receiver burial depth and the source-receiver offset. In the 0.3 km model (Figure 8A–D), the **ALW** traveltime curve is distinguishable from the **DW** only at offsets less than approximately 1.5 km, beyond which the two nearly coincide. In contrast, for the 1.0 km model (Figure 8E–H), although the **ALW** and **DW** traveltime curves are distinct across the entire offset range (up to 10 km), the actual wavelets of the two arrivals overlap at offsets greater than 1.5 km due to their proximity in arrival time.

The **ARW** is generated only when an upgoing wave impinges vertically (at normal incidence) upon the air-seawater interface. In the whole-space region directly above the HED, the fictitious EM field propagates as a TEM mode, which by definition has no vertical electric field component. Consequently, the **ARW**, which originates from this vertical propagation, is present in the horizontal component ${E^{\prime }}_{x}$ but is absent in the vertical component ${E^{\prime }}_{z}$.

The arrival time of the **ARW** is solely determined by the burial depths of the dipole source and the receivers. In a homogeneous half-space seawater model, the traveltime curve appears as a horizontal line, indicating that the **ARW** reaches all receivers simultaneously. This curve is tangential to the **ALW** traveltime curve at zero offset. For the 0.3 km model (Figure 8A,C), the **ARW** arrives at 1.11 s, which is later than the **DW** at offsets less than 0.5 km. In the 1.0 km model (Figure 8E,G), the **ARW** arrival time is 1.90 s, making it earlier than the **DW** beyond an offset of 2 km.

The damping factor accentuates the energy of early arrivals while conversely attenuating that of later arrivals. A higher frequency further increases the relative contribution of early-arriving waves. It is important to note that the shot gathers for each specific model and frequency are normalized individually by the maximum value at each receiver. Consequently, these normalized gathers should not be compared directly across different models or frequencies. In terms of true amplitude, the signal at 0.1 Hz is significantly stronger than that at 1.0 Hz.

In the 0.3 km model at 0.1 Hz, the **DW**, **ALW**, and **ARW** in the ${E^{\prime }}_{x}$ component constitute the primary arrivals within the 1–7 km offset range, with the **ARW** becoming the sole dominant wave beyond 7 km (Figure 8A). In contrast, at 1.0 Hz, the **ARW** in ${E^{\prime }}_{x}$ dominates other waves at offsets greater than 2.5 km (Figure 8C). For the ${E^{\prime }}_{z}$ component, the **DW** and **ALW** overlap, and the higher frequency enhances the relative energy ratio of the **DW** due to its earlier arrival (Figure 8B,D).

In the 1.0 km model at 0.1 Hz, the ${E^{\prime }}_{x}$ component is dominated by the **DW** and **ALW** at offsets less than 3 km. As the offset increases, the energy of the **DW** and **ALW** progressively diminishes, while that of the **ARW** concurrently strengthens, eventually making the **ARW** the predominant wave at larger offsets (Figure 8E). At 1.0 Hz, the **ARW** in ${E^{\prime }}_{x}$ becomes the dominant arrival beyond 3.5 km, although the **DW** remains the most significant component at near offsets (less than 2 km), as shown in Figure 8G. For the ${E^{\prime }}_{z}$ component, both the **DW** and **ALW** are the primary contributors to the wavefield. However, the 1.0 Hz damping factor reduces the relative energy ratio of the later-arriving **ALW** compared to the **DW** (Figure 8F,H).

Analysis of the FWD shot gathers enables the isolation of the **ARW**’s traveltime and amplitude. By strategically setting the upper integration limit in Equation (11) to encompass only the **ARW**’s arrival—where it is clearly dominant and separated—the wave can be selectively transformed back to the DFD. Applied to the 0.3 km model, an offset >2.5 km and a limit of 1.6 s are effective (Figure 8A,C). For the 1.0 km model, the required parameters are an offset >3.5 km and a limit of 2.4 s (Figure 8E,G).

In the DFD, the theoretical formula of the airwave $E_{x}^{air}\left(r\right)$ has also been given [7,16]:

(19) $$E_{x}^{air}\left(r\right)=\frac{pe^{-k\left(z+z_{0}\right)}}{2\pi \sigma r^{3}},$$

where p is the electric dipole moment, and r is the source-receiver offset in the x-direction. z and $z_{0}$ are the burial depths of the source and receiver, respectively. $k=\sqrt{i\omega \mu \sigma }$ is the wavenumber. Here, ω is the angular frequency in the DFD, μ is the magnetic permeability of free space, and σ is the conductivity of the seawater.

**Figure 9:**
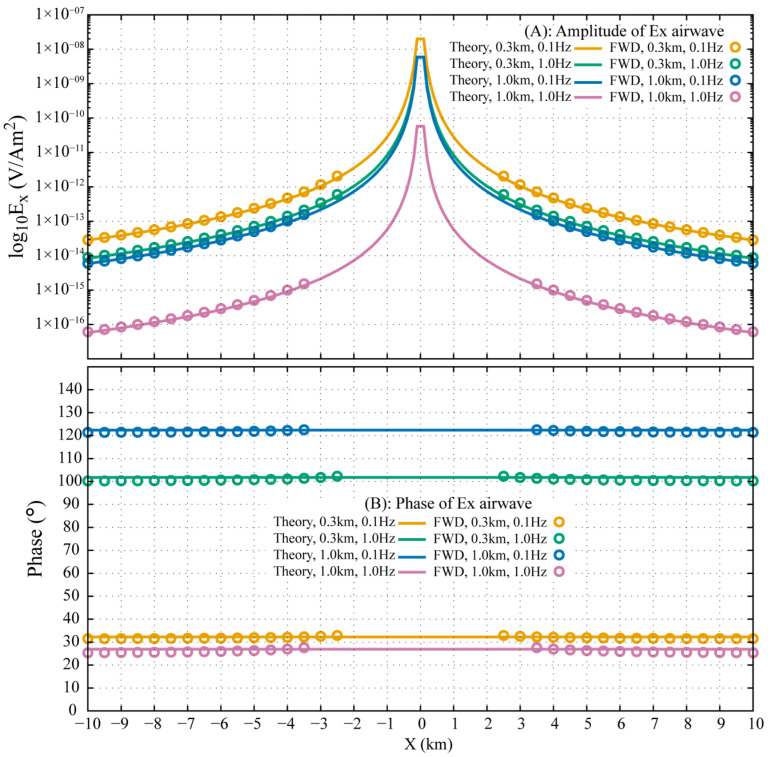
Comparison of the extracted **ARW** and the theoretical solution for $E_{x}$, showing for receiver depths of 0.3 km and 1.0 km, each evaluated at frequencies of 0.1 Hz and 1.0 Hz.

Figure 9 compares the airwaves in $E_{x}$ computed from Equation (19) with the pure **ARW** transformed from the FWD for both the 0.3 km and 1.0 km models at 0.1 Hz and 1.0 Hz. The amplitude and phase of the extracted **ARW** in the DFD show excellent agreement with the theoretical responses, with a relative amplitude error of less than 2% and an absolute phase error within 1°. This close match confirms that the **ARW** in the FWD is indeed the physical airwave observed in the DFD. The non-straight phase behavior of the **ARW** at smaller offsets indicates incomplete separation from the **DW** and **ALW** in the FWD, where residual energy from these other waves still exerts a minor influence.

As previously established, only the ${E^{\prime }}_{x}$ component exists along the axial direction of the HED. Along this particular line, the fictitious Poynting vector is zero, indicating the absence of net electromagnetic energy flow and thus no well-defined propagation direction. In contrast, if the source is a vertical electric dipole (VED), the corresponding axial direction is perpendicular to the air-seawater interface. In that case, no fictitious EM wave impinges vertically upon the interface, and consequently, the **ARW** cannot be generated.

**Figure 10:**
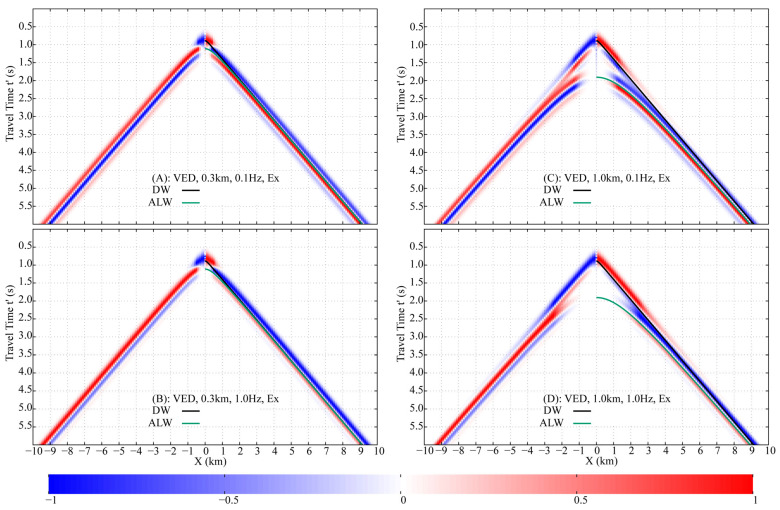
Normalized shot gathers of ${E^{\prime }}_{x}$ generated by a VED, under the same model and display configuration as Figure 8. Theoretical traveltime curves for the **DW** and **ALW** are overlaid.

Figure 10 displays normalized shot gathers of ${E^{\prime }}_{x}$ (with 0.1 Hz and 1.0 Hz damping) for the VED case in both the 0.3 km and 1.0 km models. The **DW** and **ALW** are present across all receivers, while the **ARW** is absent.

### 3.3. Airwave in Inhomogeneous Half-Space Seawater

In real-world conditions, the electrical conductivity of seawater is not uniform but varies with season, location, and depth. In practical marine CSEM surveys, seasonal and spatial conductivity variations can generally be neglected. Nevertheless, vertical conductivity variation with depth must be accounted for, as inaccuracies in the seawater conductivity model can adversely affect the inversion of subsurface properties. Key [23] evaluated inversion performance using a stratified seawater conductivity model. Their study demonstrated that jointly inverting for both seawater and subsurface conductivity—whether parameterizing the water column as a single layer or multiple free layers—yields significantly poorer results compared to inversions that utilize a known, true seawater conductivity profile and only solve for the sub-seafloor conductivity.

**Figure 11:**
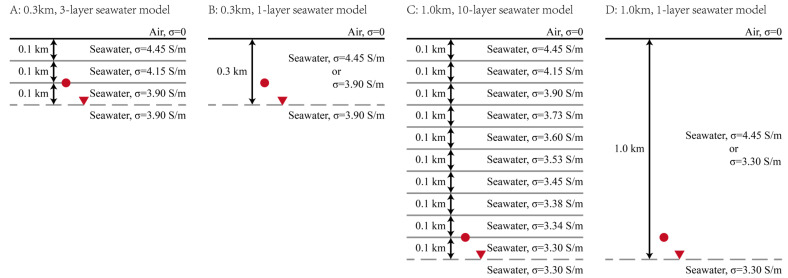
Model configurations for 0.3 km and 1.0 km seawater depths. Each depth features an inhomogeneous model (**A**,**C**) composed of 0.1 km-thick layers with distinct conductivities, and homogeneous models (**B**,**D**) assigned the maximum or minimum conductivity from the corresponding profile. The HED source (red circle) is positioned 0.1 km above the receiver (red triangle).

To represent realistic depth-dependent conductivity, stratified seawater models are constructed using statistical ocean data [24]. The 0.3 km model (Figure 11A) consists of three 0.1 km layers with conductivity decreasing downward, over an infinite layer of the lowest conductivity. The HED and receivers are located at 0.2 km and 0.3 km below the air-seawater interface. For comparison, homogeneous models (Figure 11B) are defined by merging the upper three layers and assigning the conductivity of either the top or bottom stratum. The same design approach is applied to a 1.0 km model (Figure 11C), which incorporates ten 0.1 km layers and places the source and receivers at 0.9 km and 1.0 km. Corresponding homogeneous models (Figure 11D) are constructed by merging all ten layers and selecting the top or bottom conductivity value.

**Figure 12:**
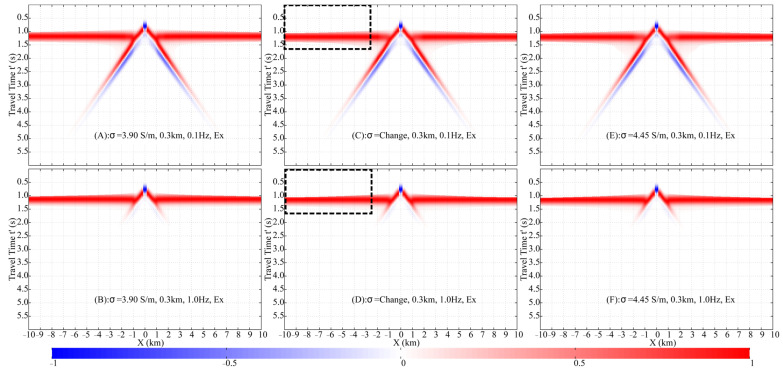
Comparison of ${E^{\prime }}_{x}$ shot gathers across homogeneous and inhomogeneous conductivity models (0.3 km depth). Models include: low-conductivity (3.9 S/m; (**A**,**B**)), layered (4.45–3.9 S/m; (**C**,**D**)), and high-conductivity (4.45 S/m; (**E**,**F**)) structures, each evaluated at 0.1 Hz (**left**) and 1.0 Hz (**right**). The black dashed rectangle is described in Figure 8.

Figure 12 shows the normalized shot gathers for the 0.3 km models with stratified and uniform conductivity at 0.1 Hz and 1.0 Hz. The uniform models have conductivities of 4.45 S/m and 3.90 S/m, respectively, while the stratified model features a conductivity gradient from 4.45 S/m to 3.90 S/m. Although the stratified model introduces more complex wave propagation paths than a simple half-space, the resulting wavefields are visually indistinguishable and remain dominated by the **DW**, **ALW**, and **ARW**. The **DW** and **ALW** overlap and exhibit nearly identical behavior across all three models. The primary difference lies in the **ARW** arrival times: the **ARW** is fastest in the 3.90 S/m uniform model and slowest in the 4.45 S/m uniform model.

**Figure 13:**
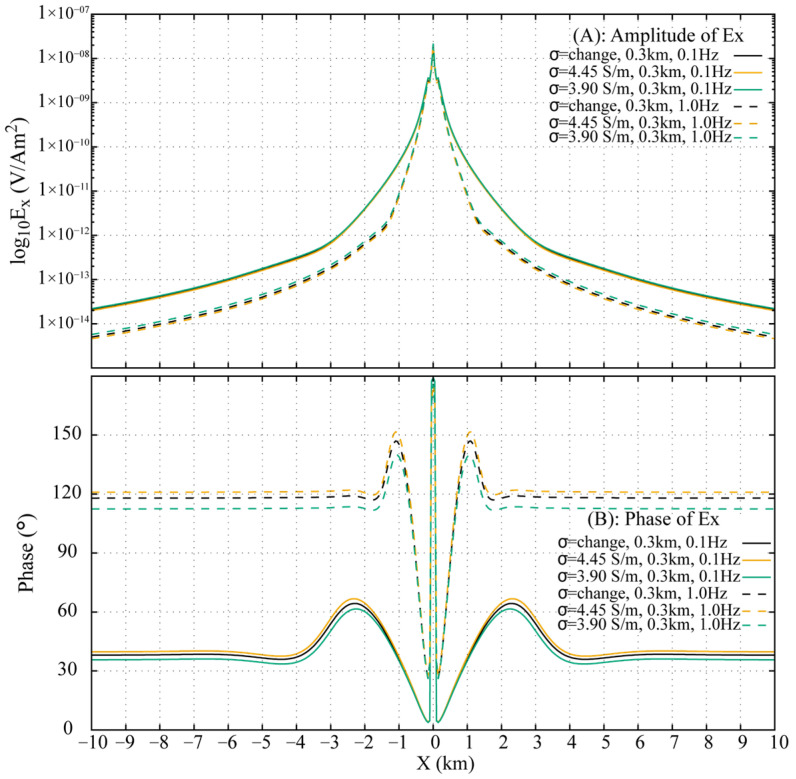
Amplitude and phase responses at 0.1 Hz and 1.0 Hz across inhomogeneous and homogeneous 0.3 km seawater models.

The fictitious field component ${E^{\prime }}_{x}$ is transformed back to the DFD, with the resulting amplitude and phase of ${E^{\prime }}_{x}$ for the three 0.3 km models at 0.1 Hz and 1.0 Hz shown in Figure 13. At offsets less than 1.0 km, the amplitude and phase responses are nearly identical across all models, which corresponds to the overlapping and indistinguishable behavior of the **DW** and **ALW** observed in the FWD shot gathers (Figure 12). Beyond 1.0 km offset, the **ARW** emerges as a dominant arrival and gradually supersedes other wave contributions. Differences in **ARW** travel times among the models are reflected in the distinguishable amplitude and phase variations of ${E^{\prime }}_{x}$ at larger offsets.

Approximating the stratified conductivity model with a homogeneous model introduces measurable inaccuracies. As summarized in Figure 13, the relative amplitude and phase errors at 0.1 Hz are 5.1% and 2.4°, respectively, for the 3.90 S/m uniform model, and 4.6% and 1.7° for the 4.45 S/m uniform model. At 1.0 Hz, the errors increase significantly: the 3.90 S/m model shows a 14.5% amplitude error and 5.5° phase error, while the 4.45 S/m model exhibits 8.9% and 3.0° errors, respectively.

Analysis of the three 0.3 km models indicates that the inaccuracies introduced by homogeneous approximations are more pronounced at 1.0 Hz than at 0.1 Hz. Replacing the stratified conductivity profile with the top-layer conductivity yields smaller errors than using the bottom-layer conductivity. While it is theoretically feasible to determine an optimal conductivity value between the top and bottom bounds to better approximate the stratified response, identifying such a value in practice remains a difficult task.

**Figure 14:**
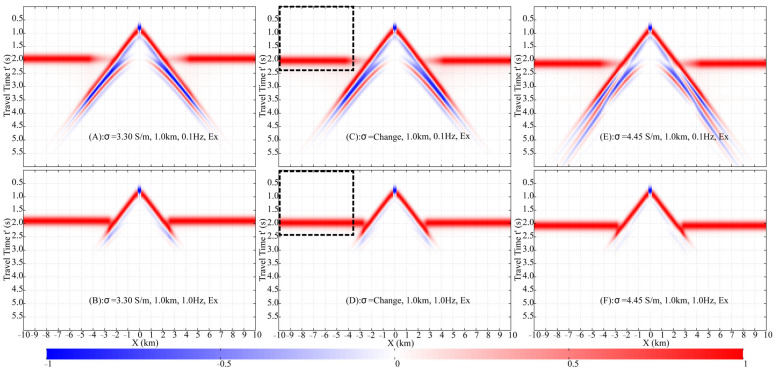
Comparison of ${E^{\prime }}_{x}$ shot gathers across homogeneous and inhomogeneous conductivity models (1.0 km depth). Models include: low-conductivity (3.3 S/m; (**A**,**B**)), layered (4.45–3.3 S/m; (**C**,**D**)), and high-conductivity (4.45 S/m; (**E**,**F**)) structures, each evaluated at 0.1 Hz (**left**) and 1.0 Hz (**right**). The black dashed rectangle is described in Figure 8.

In the 1.0 km stratified conductivity model, conductivity decreases from 4.45 S/m at the top to 3.30 S/m at the bottom (Figure 11). Analysis of the FWD results (Figure 14) reveals that at 0.1 Hz, the wavefield in the stratified model closely resembles that of the 3.30 S/m homogeneous model but differs significantly from the 4.45 S/m case. This discrepancy is attributed to the substantial conductivity contrast at the interface between the merged 1.0 km seawater column and the underlying infinite half-space in the 4.45 S/m homogeneous model, which generates additional wave conversions. At 1.0 Hz, the **DW** dominates at offsets less than 3.0 km. Notable differences are observed in the **ARW** arrival times, with the 3.30 S/m model being the fastest and the 4.45 S/m model the slowest. The greater source/receiver depths and the larger conductivity range in the 1.0 km models collectively lead to more pronounced traveltime differences for the **ARW** between the stratified and homogeneous cases.

**Figure 15:**
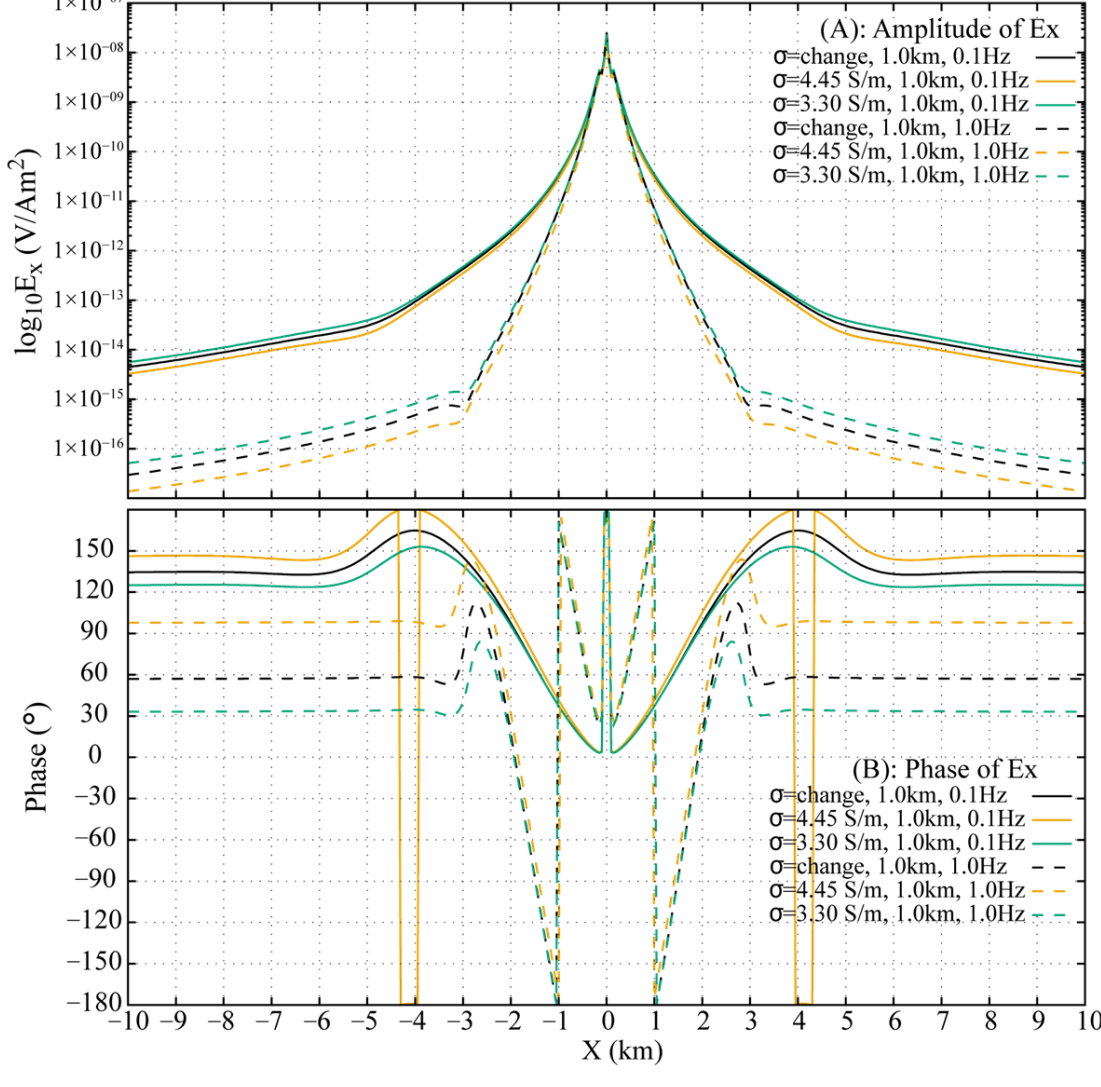
Amplitude and phase responses at 0.1 Hz and 1.0 Hz across inhomogeneous and homogeneous 1.0 km seawater models.

Model inaccuracy severely degrades the DFD response for the 1.0 km case, with errors far exceeding those of the 0.3 km models (Figure 15). For the 3.30 S/m homogeneous model, relative errors rise from 25.3% (amplitude) and 9.4° (phase) at 0.1 Hz to 72.0% and 23.8° at 1.0 Hz. Similarly, the 4.45 S/m model shows an increase from 26.5% and 11.7° to 53.7% and 40.8°, underscoring the critical impact of conductivity representation at larger scales and higher frequencies.

Although substituting the inhomogeneous seawater layers with a homogeneous layer in the 1.0 km model leads to larger relative errors, the absolute amplitude of the resulting response is significantly weaker than that in the 0.3 km model. Furthermore, the practical impact of approximating a nonuniform seawater column with a uniform conductivity in field surveys is also contingent on the conductivity structure of the subsurface and the depth of the target reservoir.

**Figure 16:**
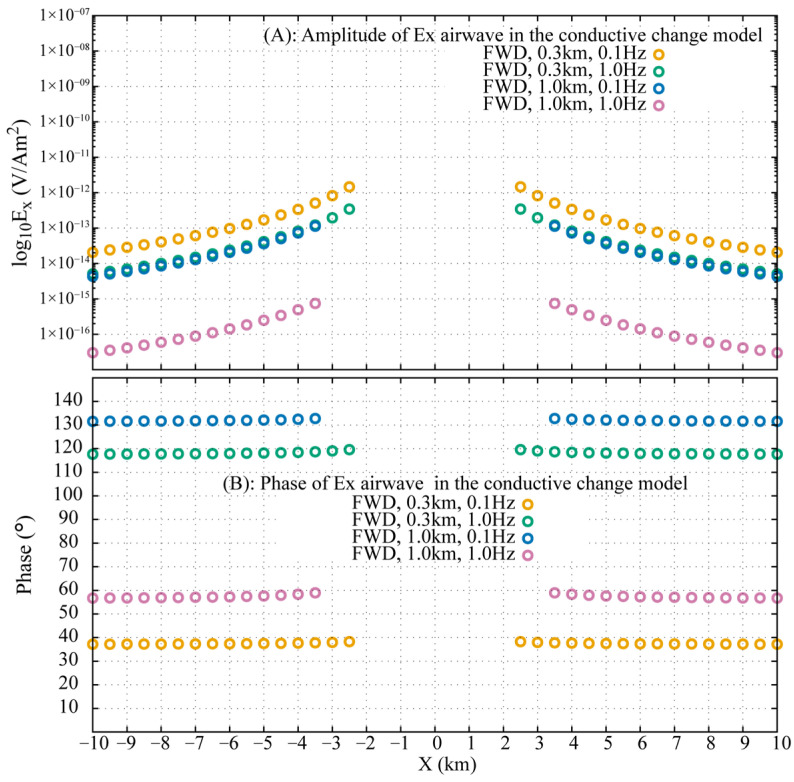
Validation of the FWD-to-DFD airwave extraction for stratified seawater models. Amplitude (**top**) and phase (**bottom**) of the extracted $E_{x}$ are shown for models of 0.3 km and 1.0 km depth at 0.1 Hz and 1.0 Hz.

When seawater conductivity is stratified, the analytical solution (Equation (19)) fails to provide an accurate airwave response. However, transforming the **ARW** from the FWD to the DFD with a properly selected integration limit provides a viable alternative. Figure 16 illustrates the successful extraction of the airwave, which is valid for offsets exceeding 2.5 km in the 0.3 km model and 3.5 km in the 1.0 km model. The method maintains phase errors below 2° even at smaller offsets where wave superposition occurs, demonstrating its robustness in realistically complex seawater environments.

## 4. Discussion: Implications for Electric Field Sensor Performance Assessment

Beyond its theoretical value for wave separation, the FWD method directly addresses the practical challenge of calibrating and assessing marine EM sensor performance.

The development of marine CSEM field sensors is a systematic and complex endeavor, typically requiring performance within a frequency range of 0.1 Hz to 30 Hz and sufficiently low self-noise levels, particularly at higher frequencies. Although the manufacturing process of Ag/AgCl electrodes is more labor-intensive compared to carbon fiber electrodes, their reversible electrochemical reaction, extremely low contact resistance, ultra-low self-potential, and stability across a broad frequency band make them the preferred electric field sensors in marine CSEM exploration [25].

Due to the industrial nature of Ag/AgCl electrodes and the precision requirements of marine electric field detection, multiple performance tests must be conducted prior to deployment. The surface morphology of the Ag/AgCl electrode is examined using Scanning Electron Microscopy (SEM) to ensure a sufficiently large specific surface area and complete coverage of AgCl. The exchange current density is measured to confirm low polarizability, faster response speed, and long-term stability. Long-term monitoring of the potential difference and potential difference drift between electrode pairs verifies consistent performance and insensitivity to environmental variations. Self-noise testing evaluates the signal-to-noise ratio of the electrode pair in weak signal detection. Active response testing involves recording the electrode pairs’ responses to artificially generated sinusoidal signals at different frequencies, verifying their accuracy in reproducing signal frequencies and the consistency of responses across multiple electrode pairs [2,26].

Active response testing represents the closest laboratory simulation of actual marine measurement conditions. However, due to the limitations of the lab environment, it cannot replicate a truly uniform electromagnetic field or precisely calculate the exact electric field strength at the electrode location. Therefore, this test primarily assesses electrode performance based on the frequency content of the recorded signals, while amplitude information offers limited evaluative value.

The deep-sea environment serves as a natural testing ground. Although the subsurface conductivity distribution may not be fully known, a reasonably accurate seawater model can be constructed through sampling and analysis. The airwave, an electromagnetic signal dependent solely on seawater conductivity and the spatial configuration of the source and receivers, has theoretical amplitude and frequency characteristics that can be modeled, numerically computed, and extracted using the FWD method proposed in this study. By comparing measured electric field signals with theoretical predictions, further evaluation and calibration of the electric field sensors’ performance can be achieved. This enhances the reliability of signals acquired by the sensors in complex marine environments and lays a solid foundation for subsequent CSEM data processing and inversion.

## 5. Conclusions

In this study, we have elucidated the propagation characteristics of fictitious electromagnetic waves excited by a horizontal electric dipole (HED) source in a whole-space model within the fictitious wave domain (FWD), with numerical results successfully transformed and validated against the diffusive frequency domain (DFD). For the half-space seawater model, our findings demonstrate that the airwave is generated when vertically upgoing transverse electromagnetic (TEM) signals interact with the air–seawater interface, producing a distinct refracted component. In contrast, no airwave is observed in the vertical electric dipole (VED) case, which aligns with the absence of net energy propagation along the dipole axis.

Within the FWD representation of the half-space model, the FWD approach successfully visualizes the propagation process of the airwave, enabling clear identification of three primary wave types: the Direct Wave (**DW**), the Air Reflection Wave (**ALW**), and the Air Refracted Wave (**ARW**). Using seawater models with depths of 0.3 km and 1.0 km, we illustrate clear traveltime differences among these waves, enabling effective separation of the **ARW**—the airwave in the DFD—at sufficiently large offsets. By judiciously selecting the upper limit for the integral transformation from FWD to DFD, the pure airwave can be accurately extracted and reconstructed in the DFD, showing excellent agreement with theoretical responses.

Furthermore, this study highlights that approximating stratified or inhomogeneous seawater conductivity with a uniform layer can introduce substantial inaccuracies in simulated responses. The proposed FWD-based extraction method remains effective even under such complex conductivity structures, offering a reliable approach for simulating pure airwave responses in realistic marine environments. The investigation into the propagation mechanism, visualization, and extraction of the pure airwave provides a theoretical foundation for the design of marine CSEM sensors and other applications relying on electromagnetic fields excited by electric dipoles in seawater.

Application of the FWD method to air wave analysis reveals its unique capability to characterize EM fields through fictitious wave propagation parameters (paths, travel times, and energy) and to extract specific EM signals. This provides a precise tool for the detailed study of low-frequency EM behavior in marine geo-electrical models. With further development of numerical methods and computing power, the FWD approach can address complex 3D scenarios, thereby contributing to next-generation marine CSEM technology in electrode development, data processing, and inversion.

## Data Availability

The original contributions presented in this study are included in the article. Further inquiries can be directed to the corresponding author.
